# Study on the Properties of FEVE Modified with Ag_2_O/OH-MWCNTS Nanocomposites for Use as Adhesives for Wooden Heritage Objects

**DOI:** 10.3390/molecules29061365

**Published:** 2024-03-19

**Authors:** Gele Teri, Cong Cheng, Kezhu Han, Dan Huang, Jing Li, Yujia Luo, Peng Fu, Yuhu Li

**Affiliations:** 1 Engineering Research Center of Historical Cultural Heritage Conservation, Ministry of Education, School of Materials Science and Engineering, Shaanxi Normal University, Xi’an 710119, China; terigelesnnu@163.com (G.T.); congcheng2017@snnu.edu.cn (C.C.); hankekezhu@126.com (K.H.); hdansnnu@163.com (D.H.); 2 Shandong Museum, Jinan 250014, China; lijing9669@126.com; 3 Shaanxi Institute for the Preservation of Culture Heritage, Xi’an 710075, China

**Keywords:** Ag_2_O/OH-MWCNTS-FEVE, adhesives for wooden artifacts, bonding strength, antibacterial

## Abstract

The durability of wooden heritage objects and sites can be affected by external environmental factors, leading to decay, cracking, and other forms of deterioration, which might ultimately result in significant and irreversible loss. In this study, a FEVE resin was modified with Ag_2_O/OH-MWCNTS (MA), denoted as MAF, where three concentrations were prepared using in situ precipitation, and the resulting composite adhesive was characterized by a high viscosity and effective bacteriostatic properties, demonstrating a better viscosity and thermal stability, as well as antibacterial properties, than pure FEVE resin. The results show that MAF adhesives present good thermal stability, as evidenced by a lower mass loss rate following treatment at 800 °C compared to the pure FEVE resin. At a consistent shear rate, the viscosity of MAF demonstrates a notable increase with the proportion of MA, which is better than that of FEVE. This suggests that the nano-Ag_2_O particles in MA act as physical crosslinking agents in FEVE, improving the viscosity of the composite adhesive MAF. The adhesion strength between MAF and wood exhibits a similar trend, with wooden samples showing higher shear strengths as the proportion of MA increases in comparison to FEVE. Simultaneously, the antibacterial effects of the MAF adhesive exceeded 1 mm for *Trichoderma*, *Aspergillus niger*, and *white rot fungi*. The antibacterial activity of the MAF adhesive exhibited a direct correlation with the concentration of Ag_2_O/OH-MWCNTS, with the most pronounced inhibitory effect observed on Trichoderma. The MAF adhesive demonstrates promising prospects as an adhesive for wooden heritage artifacts, offering a novel approach for the rapid, environmentally friendly, and efficient development of composite adhesives with superior adhesive properties.

## 1. Introduction

Wood, as a readily accessible and easily processed natural resource, has been utilized throughout history in various aspects of human life. In ancient civilizations, when alternative materials were scarce, wood was extensively employed for the construction and ornamentation of historical sites [1,2]. Across different cultures and time periods, a plethora of intricate wooden artifacts, crafts, and structures have been preserved as cultural relics, frequently documented in historical records [3]. During the middle Paleolithic Age (~400,000 years ago) various heritage artifacts and sites, such as sculptures, lacquered wooden ware, bridges, defense weapons, wooden buildings of the Imperial Palace, carriages, ships, and other items, were created [4,5]. These wooden cultural heritage items serve as valuable materials for research on ancient history, culture, and living standards, and therefore possess significant historical and artistic value [4,6,7,8]. Furthermore, these artifacts and sites, which cannot be replicated, exemplify the ingenuity and resourcefulness of humanity.

Wood is a natural bio-composite whose physical and chemical properties change as a result of various factors during the preservation process, leading to varying degrees of degradation. The loss of mechanical stability due to degradation significantly impacts the lifetime and structure of wooden cultural relics [9,10,11,12,13,14]. Cellulose, hemicellulose, and lignin are the primary components of wood, where the free hydroxyl groups are abundant. The fluctuation of temperature and humidity in the preservation environment allows for the free absorption and release of water vapor by the hydroxyl groups in wood, leading to changes in the wood’s humidity. The synergistic effect caused by these factors can result in alterations to the size of the wood and the development of cracks [15,16,17], which might ultimately result in the deterioration and loss of wooden cultural relics, leading to significant and irreparable losses in historical research and cultural heritage. Hence, the preservation of wooden cultural artifacts is imperative and significant, necessitating the advancement of appropriate adhesives for such objects [18].

According to the definition by Encyclopedia Britannica, an adhesive refers to any substance that is capable of holding materials together [19]. The history of adhesives is closely associated with human history, becoming one of the most extensively used materials in early human societies with the advent and evolution of composite tools during the Mesolithic era. Adhesives, initially employed for hafting, were found to have wide-ranging applications in painting, decoration, lacquerware, bows and arrows, construction, and pottery repair [20,21,22,23,24,25]. In ancient times, a variety of adhesive materials were utilized, including animal glues, eggs, casein, blood, plant resins, gums, starch, bitumen, tar, wax, and oils. With technological advancements, the diversity of adhesives has expanded, including various organic and inorganic materials. Common adhesives used in the conservation of wooden cultural heritage artifacts include natural adhesives such as casein, rabbit skin glue, fish glue, waxes, oils, and natural resins, as well as synthetic adhesives like the copolymers of methyl methacrylate and ethyl acrylate (Paraloid B72) and polyvinyl acetate emulsions (Ravemul M18—Vinavil). However, these adhesives have their respective advantages and disadvantages. For instance, animal glues are natural, non-toxic, fully reversible, and exhibit excellent adhesion to wooden surfaces without staining, but can be susceptible to microbial growth.

**Figure 1:**
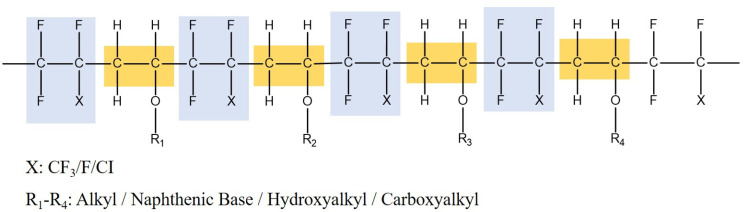
Molecular formula of FEVE.

A lot of attention has been paid to FEVE fluorocarbon [26,27] resin in the field of coating research and development owing to its unique structural properties (Figure 1). Currently, research on FEVE primarily revolves around the development of coatings and the regulation of their performance. The high bond energy of the C-F fluorine-containing bond in fluorocarbon resin at ~485 kJ/mol is attributed to the large electronegativity of fluorine atoms [28]. This results in a polymer with significant repulsive forces between adjacent fluorine atoms, leading to a high stability and exceptional aging resistance. Additionally, FEVE can be cured and shaped at room temperature, showing a low surface energy, strong adhesion properties, and other favorable characteristics, making it a suitable option for wood adhesives. However, challenges such as low tensile strength and inadequate bacteriostatic properties still exist.

Wood is prone to microbial degradation, particularly by fungi, leading to a decrease in mechanical stability [29]. Microbial corrosion can affect both wood itself and adhesive materials, indicating the importance of antibacterial properties in protecting wooden cultural artifacts from wood-rot fungi [30,31]. In recent years, antibacterial nanomaterials have emerged as promising candidates for antimicrobial applications within the field of chemical antibacterial technologies, owing to their expansive specific surface area and distinctive chemical and physical characteristics [32,33]. Numerous nanomaterials, such as ZnO, CuO, TiO_2_, and Ag_2_O, have demonstrated exceptional antibacterial properties and are utilized as antibacterial agents [34,35,36,37]. Notably, silver oxide nanoparticles offer advantages such as ease of production, cost-effectiveness, and potent antibacterial and antifungal activity. Silver oxide antibacterial nanoparticles (NPs) are commonly utilized for their notable antibacterial efficacy against a diverse range of bacteria [38,39,40], leading to their utilization as a preservative in various medical devices, food packaging, and environmental purification procedures [41,42,43]. Nonetheless, nano Ag_2_O is hindered by inadequate dispersion. Then, carbon nanotubes (CNTs) began to be used as a novel class of nanomaterials following their inception in 1991 [44], offering substantial application benefits across numerous scientific and technological domains [45,46,47]. Hydroxylated multi-walled carbon nanotubes (OH-MWCNTS) are a novel carbon-based material with superior properties such as increased weather resistance, environmental compatibility, corrosion resistance, lightweight construction, expansive surface area, excellent thermal stability, and robust biological compatibility when compared to similar materials. Currently, hydroxylated MWCNTS are extensively utilized in chemical reactions, environment protection, biological classification and conversion, electrochemical energy storage, and other related fields [48,49,50,51]. Important findings have been reported based on studies of MWCNTs/FEVE composite coatings [52]. MWCNTs can be uniformly distributed within the composite coating regardless of the amount. As the content of MWCNTs increases in the coating, both the glossiness and the static friction coefficient of the coating significantly decrease. Also, the surface of the coating becomes rougher, and the composite coating exhibits both hydrophobic and oleophobic properties. Adding a small amount of MWCNTs to the coating can greatly improve the conductivity of the MWCNTs/FEVE composite coating. The incorporation of hydroxyl groups may enhance the interaction between MWCNTs and FEVE resin, thereby improving the load transfer efficiency and interface bonding strength of the composite. The exceptional chemical properties and weathering resistance of FEVE resin make it ideal for the conservation of outdoor heritage objects. The addition of OH-MWCNTs can further enhance these properties, leading to a composite which is suitable for harsh environmental conditions. The introduction of OH-MWCNTs may also affect the surface properties of the composites, including roughness and hydrophobicity/hydrophilicity.

Based on the above discussion, this study involved the preparation of a stable and low-cost composite material consisting of Ag_2_O-decorated hydroxyl multi-wall carbon nanotubes, i.e., Ag_2_O/OH-MWCNTS (MA), using an in situ precipitation method. This composite material was then mixed with a FEVE resin to prepare a durable and bacteriostatic wooden adhesive, i.e., Ag_2_O/OH-MWCNTS-FEVE (MAF), which was subsequently subjected to the exploration of variolous properties. The structural and performance characteristics of the resulting nanomaterials were analyzed using techniques such as infrared spectroscopy, X-ray diffraction, and SEM spectroscopy. The thermal stability and viscosity of the MAF wood adhesive were assessed, while the shear strength and antibacterial properties of the wood adhesive were investigated using a universal tensile testing machine and an antibacterial zone test. Comparative analysis revealed that the modified MAF exhibited superior viscosity and bacteriostatic properties compared to the original FEVE, suggesting a promising application potential for the composite material.

## 2. Results and Discussion

### 2.1. X-ray Diffraction Analysis

**Figure 2:**
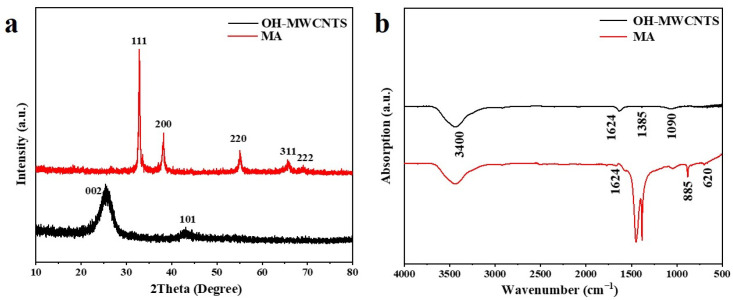
(**a**) XRD spectra and (**b**) FTIR spectra of the OH-MWCNTS and MA.

Each crystalline has a distinct atomic arrangement and exhibits a characteristic X-ray diffraction pattern, serving to identify its crystal structure. X-ray diffraction (XRD), a nondestructive method, is commonly employed to determine the crystal structure and purity of nanoparticles [53]. The XRD patterns of the nanocomposites Ag_2_O/OH-MWCNTS and OH-MWCNTS are shown in Figure 2a. The 26.1o and 42.9o in the XRD patterns of OH-MWCNTS correspond to the reflections of the surfaces of hexagonal graphite (JPCDS No. 75-1621) (002) and (101). The diffraction angles (2θ) at 26.2°, 32.9°, 38.1°, 55.2°, 65.7°, and 69.1°, correspond to the groups of Ag_2_O lattice planes (cubic structure) (110), (111), (200), (220), (311), and (222), respectively [JCPDS No.76-1393]. The presence of a prominent intensity peak in the (111) plane suggests a well-ordered arrangement of lattice atoms [54]. Furthermore, the absence of peaks corresponding to other substances in the X-ray diffraction pattern suggests a high concentration of Ag_2_O within the nano-composite Ag_2_O/OH-MWCNTS.

### 2.2. Infrared Absorption Spectroscopy Analysis

Fourier transform infrared spectroscopy (FTIR) spectroscopy was used to investigate the characteristics of the functional groups of Ag_2_O/OH-MWCNTS and OH-MWCNTS as shown in Figure 2b. The broad band at 3400 cm^−1^ indicates the O–H stretching vibrations of the hydroxyl groups corresponding to H-bonded alcohols and also to intramolecular H bonds, which confirms the existence of the O-H group on the surface of the OH-MWCNTs [55]; a band at 1624 cm^−1^ is attributed to the stretching vibrations in the carbon backbones of OH-MWCNTs. Based on findings from previous studies, metal–oxygen stretching frequencies typically present within the range of 500 to 600 cm^−1^ [56]. In Figure 2b, the peak shown at 620 cm^−1^ is attributed to the Ag-O vibration of Ag_2_O [57]. The absorption peak at 885 cm^−1^ corresponds to the Ag-O bond [58], while the peak at 1271 cm^−1^ is notably strong, indicating a high Ag_2_O content in Ag_2_O/OH-MWCNTS. These results are consistent with the XRD data, providing further evidence for the formation of Ag_2_O/OH-MWCNTS.

### 2.3. SEM Analysis

**Figure 3:**
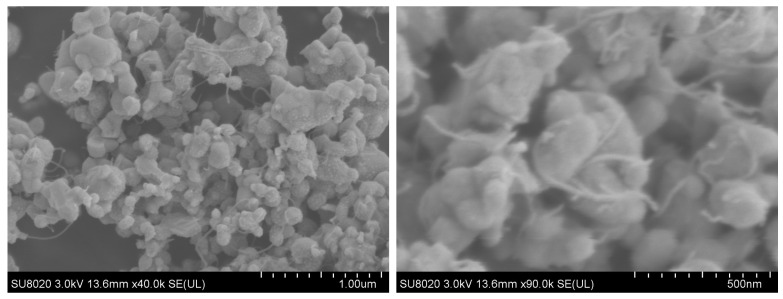
SEM images of the Ag_2_O/OH-MWCNTS with different magnifications.

Figure 3 presents scanning electron microscope (SEM) surface morphology images of the nano-composites (Ag_2_O/OH-MWCNTS) at various magnifications. The images reveal a uniform near-spherical nanobeam of Ag_2_O with a diameter of 19–59 nm, with OH-MWCNT dispersed among the nanobeams, which is consistent with findings from FTIR and XRD analyses. Typically, smaller particle sizes are more beneficial for improving antibacterial activity, which is due to the reduced particle size; a greater number of particles can readily adhere to the bacterial cell membrane surface, facilitating successful cell penetration and subsequent destruction of the cell’s physiological functional groups [59]. The SEM images indicate that the in situ synthesis of nano-composites (Ag_2_O/OH-MWCNTS) offers a straightforward and efficient method to achieve the requisite size specifications for Ag_2_O.

### 2.4. Thermal Stability Analysis

**Figure 4:**
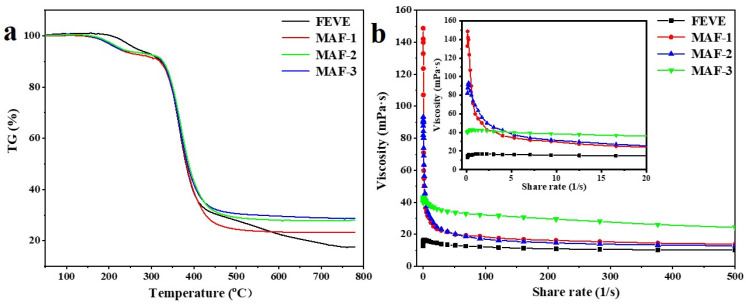
(**a**) TG spectra of the MAF adhesive with different concentrations; (**b**) viscosity–shear rate diagram of the MAF adhesive with different concentrations.

The thermal properties of the FEVE and MAF composites were examined using thermogravimetric (TG) analysis, and the results are presented in Figure 4a. The DTG curves exhibit a general similarity among the samples. The initial peaks observed in the TG curves of MAF-1, MAF-2, and MAF-3 between 150 and 220 °C are attributed to the evaporation of surface and interlayer moisture. Subsequently, a second peak is observed between 330 and 470 °C, indicating the degradation of oxygen-containing functional groups on the surface (OH-MWCNTS) and the degradation of chemical bonds within the FEVE resin. Given the low content of MWCNT [60], no weight loss induced by MWCNT oxidation was found at temperatures ranging from 600 to 800 °C. Mass losses of 76.7%, 72.1%, and 69.9% were observed for MAF-1, MAF-2, and MAF-3, respectively, in the temperature range of 200–800 °C. In comparison, the FEVE resin presents a mass loss of 82.5%, indicating a gradual decrease in mass loss with increasing concentrations of Ag_2_O/OH-MWCNTS. The incorporation of Ag_2_O/OH-MWCNTS resulted in the enhanced thermal stability of FEVE.

### 2.5. Viscoelastic Measurement

The viscosity of the adhesive plays a crucial role in determination of its permeability and hydrophilicity on the substrate surface, as well as its adhesion properties [61]. Figure 4b shows the change in apparent viscosity of the composite adhesive MAF with different concentrations of MA added as the shear rate increases. It is evident that the shear thinning effect of FEVE viscosity is relatively small when the shear rate increases. FEVE possesses a multitude of C-F chemical bonds, with the C-F bond energy reaching a remarkable 485.6 kJ/mol, resulting in minimal polarity and a stable molecular structure. From a structural perspective, the FEVE structure comprises three Fs, forming a spiral-like three-dimensional arrangement that tightly encircles each C-C bond within the molecule, filling in the gaps between C-C bonds, thereby ensuring maximum structural integrity and tightness [28]. Therefore, the viscosity of FEVE changes little with the increase in the shear rate. When the shear rate is constant, the apparent viscosity of MAF increases with the increase in the MA concentration. Upon investigation, nano-Ag_2_O was immobilized on the surface of OH-MWCNTS, leading to an enhanced specific surface area of the material. Nano-sized Ag_2_O particles play a role as physical crosslinking points in FEVE, thereby increasing the viscosity of the composite adhesive M [28,62].

MAF-1 and MAF-2 rapidly decrease in the low shear rate region, gradually decrease in the high shear rate region, and reach the viscosity of infinite shear rate. When the shear rate of MAF-1 and MAF-2 was increased from 0.1 to 20 s^−1^, the viscosity decreased significantly, indicating that the composite adhesive exhibited shear thinning behavior and had pseudoplastic properties. This is due to the low content of MA, which results in fewer physical crosslinking points, leads to unstable intermolecular forces and intermolecular interactions that are easily disrupted under high shear rates. The content of Ag_2_O/OH-MWCNTS in MAF-3 forms a sufficient number of physical cross-linking points, so the viscosity changes slightly with the increase in shear rate. Due to the effect of MA on the viscosity of the composite adhesive MAF, this will affect the adhesion between the adhesive and the substrate, so we verified its bonding strength.

### 2.6. Analysis of Adhesive Bonding Shear Strength

**Figure 5:**
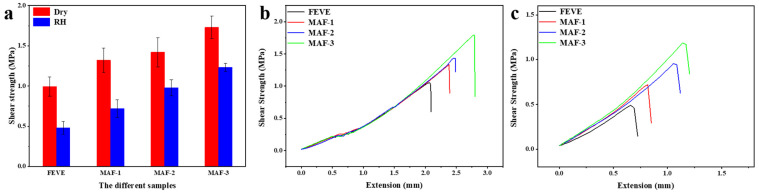
The shear strength of the wooden samples bonded with the MAF adhesives (**a**); stress–displacement curves for the wooden samples bonded using adhesives after drying (**b**) and after high RH degradation (**c**).

The cohesion and adhesion of MAF play crucial roles as the adhesive in composite nanomaterials. When the water-based FEVE interacts with the surface of wood, it exhibits permeability characteristics. And, with the addition of MA, the viscosity of the composite adhesive MAF increases, thus reducing the permeability of MAF on the surface of porous wooden materials. The binding force necessitates the formation of a continuous crystalline phase structure, with the strength of the bond being largely dependent on the molecular attraction between the adhesive and the substrate surface [63]. Figure 5 shows the shear strength and stress–strain curve changes of wooden panels using FEVE, MAF-1, MAF-2, and MAF-3 adhesives. The shear strength values for the wood fixed using FEVE, MAF-1, MAF-2, and MAF-3 were 0.994 MPa, 1.32 MPa, 1.42 MPa, and 1.73 MPa (Table 1), respectively. After 5 days of degradation, the bonded wooden samples exhibited a notable decrease in shear strength compared to the samples before degradation. The reductions in shear strength after degradation were measured at 51.71%, 45.45%, 30.99%, and 28.90% for wooden samples bonded with FEVE, MAF-1, MAF-2, and MAF-3, respectively. Although the shear strength of the FEVE adhesives decreased under high relative humidity, the incorporation of MA led to a notable increase in the adhesive strength of MAF. With the increase in MA, the shear strength of plywood increased, in both the dry and after high RH degradation. As the content of MA increases, the shear strength of the wooden panel increases. The results show that the addition of MA can improve the bonding properties between FEVE and the wooden panel. It is reported that carbon nanotubes added as fillers in fluorocarbon coatings can form functional networks and improve the compactness of the coating. Nano-sized Ag_2_O is deposited on the surface of OH-MWCNTS, increasing the specific surface area of OH-MWCNTS, and the number of physical cross-linking points [64,65,66]. Small-size inorganic Ag_2_O nanoparticles can effectively fill the structural micropores generated during the curing process. Therefore, the bonding force between MAF and the wooden panel is increased. The stress–displacement curve directly reflects the properties of the material itself. As the content of MA increases, the stress on the composite adhesive MAF increases. Under the same stress, the displacement of FEVE is greater than that of MAF-1, MAF-2, and MAF-3. The results showed that the addition of MA increased the brittleness of the composite adhesive MAF material, which was related to the formation of a dense coating.

### 2.7. Antimicrobial Effects of Ag_2_O/OH-MWCNTS-FEVE Adhesives

**Figure 6:**
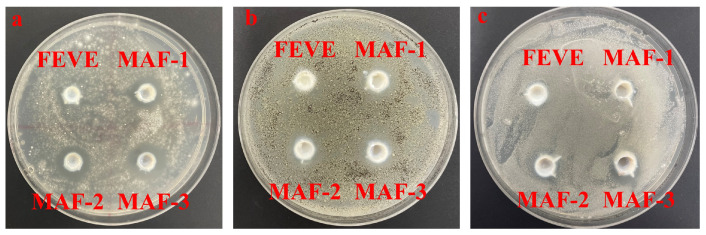
Inhibitory effects of different concentrations of MAF on (**a**) *Trichoderma*, (**b**) *Aspergillus niger*, and (**c**) *white rot fungi*.

Figure 6 present the results of a comparative analysis of the antibacterial activity of the MAF-1, MAF-2, MAF-3, and FEVE adhesives, where all of the MAF adhesives exhibited inhibitory effects on three types of molds, i.e., *Trichoderma*, *Aspergillus niger*, and *white rot fungi*, where the best inhibitory effects were presented for Trichoderma (Figure 6). The images indicate that the inhibitory effects of MAF were dose-dependent, as the size of the inhibitory halo is directly proportional to the concentration of Ag_2_O/OH-MWCNTS, with values exceeding 1 mm considered effective. According to established criteria, any antibacterial agent producing an inhibitory zone larger than 1 mm is classified as “good” [67]. Consequently, the incorporation of Ag_2_O/OH-MWCNTS resulted in a notable enhancement of the antibacterial efficacy of FEVE adhesives.

## 3. Materials and Methods

### 3.1. Materials

AgNO_3_ and NaOH were purchased from Sinopharm Chemical Reagent Co., Ltd. OH-MWCNTS was purchased from Xianfeng nanomaterial Technology Co., Ltd. (Jiangsu, China), Potato Dextrose Agar (PDA) was purchased from Aobox Biotechnology, *Trichoderma*, *Aspergillus niger*, and *white rot fungi* were purchased from Chinese Academy of Forestry (Beijing, China).

### 3.2. Preparation of Ag_2_O/OH-MWCNTS-FEVE (MAF) Adhesives and Plywooden Samples

A total of 100 mg of OH-MWCNTs was dispersed in a 50 mL solution of 0.5 mol/L AgNO_3_ (ethanol: deionized water = 1: 1) with sonication at a frequency of 10,000 Hz for 30 min. Subsequently, a 0.5 mol/L NaOH solution was incrementally added to the suspension of OH-MWCNTs/AgNO_3_ in ice, with continuous stirring. Then, the resulting products were extracted, washed with deionized water and ethanol, and dried at 75 °C for 12 h, labelled as Ag_2_O/OH-MWCNTs (MA), and stored in a desiccator until further use. 100 μg/mL, 200 μg/mL, and 300 μg/mL Ag_2_O/OH-MWCNT-FEVE (MAF) were prepared and labelled as MAF1, MAF2, and MAF3, respectively. These solutions were subsequently sealed and stored for subsequent use.

The FEVE was diluted with ultrapure water to obtain a 50% aqueous solution and was labeled as FEVE.

### 3.3. Characterization

#### 3.3.1. X-ray Diffraction Analysis

We weighed and characterized 10 mg Ag_2_O/OH-MWCNTS and OH-MWCNTS powders using a high-resolution X-ray diffractometer (XRD, Smart Lab 9, Rigaku, Japan), with test conditions for Cu Kα ray (λ = 1.54056 A), a 2θ range of 20°–80°, acceleration voltage of 45 kV, tube current of 200 mA, and scanning speed of 5°/min.

#### 3.3.2. Infrared Absorption Spectroscopy

We used 0.2 mg Ag_2_O/OH-MWCNTS and OH-MWCNTS powders to prepare KBr pellets, and FTIR spectra were collected using the Fourier transform infrared (FTIR) spectrometer (Nicolet iS10, Thermo Fisher Scientific, Waltham, MA, USA). The spectral range was set between 4000 and 400 cm^−1^, with a resolution of 4 cm^−1^, and both the sample and background were scanned 64 times. The molecular structures of the samples were identified through the characteristic peaks of various functional groups in the spectra.

#### 3.3.3. Scanning Electron Microscopy

The particles of Ag_2_O/WCNTS were attached to the sample stage with the conductive adhesive, and the samples were sprayed with gold on the surface. Finally, the micro-morphology of the samples was obtained using a Field emission scanning electron microscope (SU8020, Hitachi Company, Japan).

#### 3.3.4. Thermogravimetric Analysis

The thermal analysis of the three Ag_2_O/OH-MWCNTS-FEVE adhesive samples (MAF-1, MAF-2, MAF-3) was performed with the Thermogravimetric analyzer (Themys One, Setaram, France) to evaluate its thermal stability. During the test, N_2_ was used as the protective gas, the temperature range was set at 30–800 °C, and the heating rate was 20 °C/min.

#### 3.3.5. Viscoelastic Measurement

The three Ag_2_O/OH-MWCNTS-FEVE adhesive samples (MAF-1, MAF-2, MAF-3) were used and tested using the rheometer (MCR 302, Anton Paar, Austria). Rotors of PP50 were selected and 2 mL of the solution was taken with pipettes and placed on the test stage. The viscosity of the corresponding sample was measured in the shear rate range of 0.1–500 s^−1^, and all measurements were made at a constant temperature of 23 °C.

#### 3.3.6. Shear Strength

**Figure 7:**
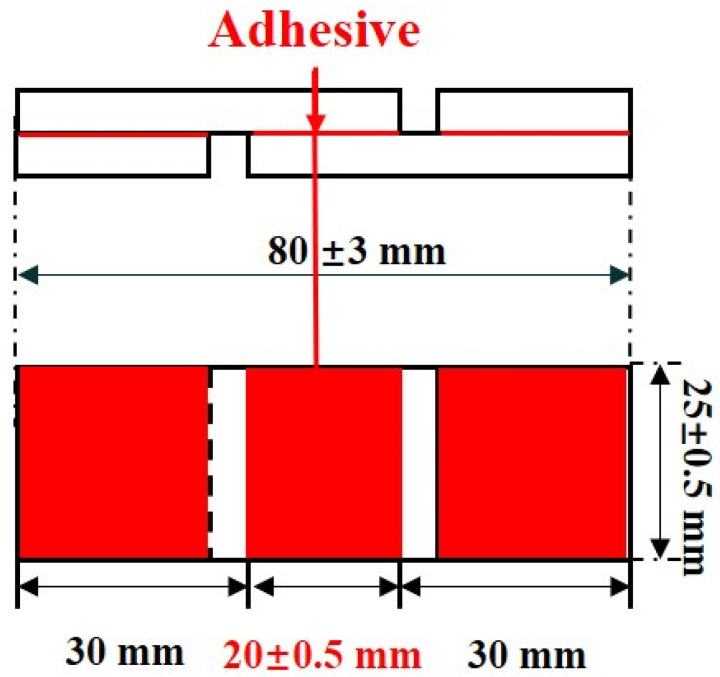
Schematic diagram of the test specimen used for shear strength measurement.

**Figure 8:**
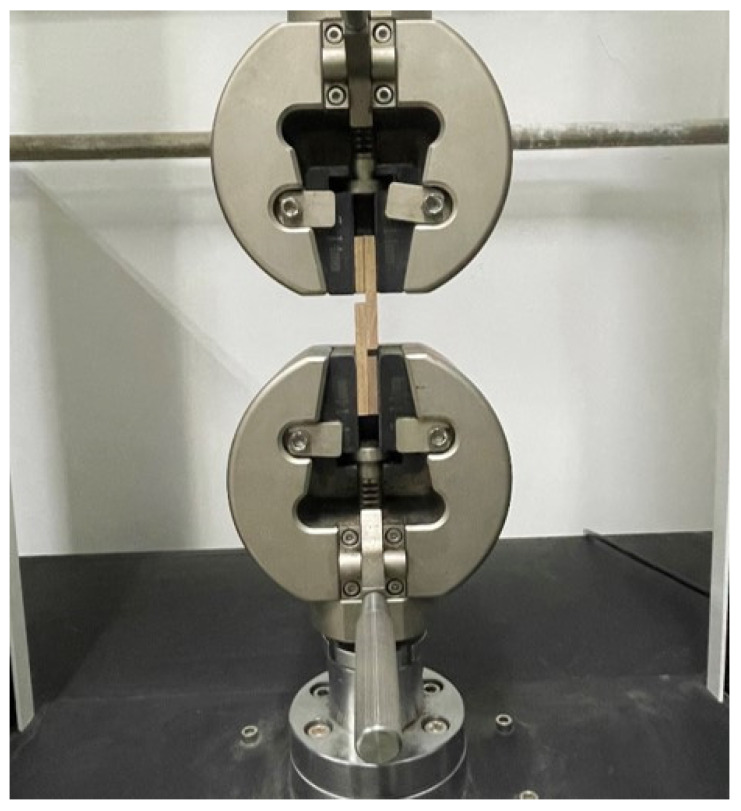
Positioning of a pair of wooden samples on The Universal Material Testing Machine.

According to the standard ISO 6237:2003, adhesive should be applied to the surface of a wooden block sample with a coating area of 500 mm^2^ (25 mm × 20 mm). Another wooden block of the same size should then be placed on top of the adhesive area, ensuring complete coverage, and clamped to secure. The assembled sample should be left at room temperature (23 ± 2 °C, relative humidity 50% ± 10) for 7 days. The assembled samples were used to measure the dry shear strength, or degradation under specific environmental conditions of 90% ± 5 relative humidity and 23 ± 2 °C for a duration of 5 days and tested with a high RH degradation shear strength test afterwards. The Universal Material Testing Machine (Kc-136pc, Xi’an Kaicheng Technology Co., Ltd., Xi’an, China) was utilized at a speed of 20 N/min for the test. This test was performed 10 times for each sample, and the average was calculated and used. The schematic diagram of the specimen for shear strength measurement is illustrated in Figure 7. Positioning of wooden samples on The Universal Material Testing Machine in Figure 8.

#### 3.3.7. Antifungal and Antibacterial Effects of Ag_2_O/OH-MWCNTS-FEVE Adhesives

*Aspergillus niger*, *Trichoderma*, and *white rot fungi* were cultured in PDA inclined tube, then fungal spores were isolated with 0.05% Tween 20 and filtered with gauze. After counting mold spores with a blood cell counting plate, the spore suspension was diluted with phosphate-buffered saline (PBS) until 1 × 10^5^ colony forming untis (CFU) was obtained. Then, 100 μL of 1 × 10^5^ CFU/mL spore suspension was evenly applied to the PDA medium, and the medium was evenly divided into 4 parts along the center. An Oxford cup was placed in the center of each part, and 100 μL FEVE solution was added to the control group, and 100 μL MAF-1, MAF-2, MAF-3 solutions (100 μg/mL, 200 μg/mL, 300 μg/mL) were added to the treatment group successively, and cultured in an incubator at 25 °C with 80% RH for 48 h. The sizes of the fungal inhibition zones were observed and the images were collected.

## 4. Conclusions

Overall, a wood adhesive with elevated viscosity and effective bacteriostatic properties was synthesized through a straightforward method utilizing FEVE resin as the primary material given its superior viscosity and thermal resilience. Furthermore, the Ag_2_O/OH-MWCNTS was prepared using an in situ precipitation technique, followed by the preparation of a composite adhesive by blending it with FEVE resin, denoted as MAF. The MAF adhesive displayed an exceptional thermal stability, with a lower mass loss rate than the original FEVE resin following treatment at 800 °C. At a consistent shear rate, the viscosity of MAF demonstrates a notable increase with the proportion of MA being higher than that of FEVE. This suggests that the nano-Ag_2_O particles present in MA act as physical crosslinking agents in FEVE, enhancing the viscosity of the composite adhesive MAF. The adhesion strength between MAF and wood exhibits a similar trend, with wooden samples displaying higher shear strengths as the proportion of MA increases in comparison to FEVE. Additionally, the antibacterial effects of MAF adhesive were observed to be greater than 1mm for *Trichoderma*, *Aspergillus niger*, and *white rot fungi*. We also found that the antibacterial properties of the MAF adhesive were directly correlated with the concentration of Ag_2_O/OH-MWCNTS, leading to the most effective inhibition of *Trichoderma*. Consequently, the MAF adhesive demonstrates significant promise for use as an adhesive for wooden artifacts, offering a novel approach for the rapid, environmentally friendly, and efficient preparation of composite adhesives with superior adhesion capabilities.

## Figures and Tables

**Table 1:** Shear strength data for the wooden samples.

Adhesives	FEVE	MAF-1	MAF-2	MAF-3
Dry strength (MPa)	0.99	1.32	1.42	1.73
RH strength (MPa)	0.48	0.72	0.98	1.23

## Data Availability

The data presented in this study are included in the article.
